# Tolerance for Housing Unaffordability among Highly Skilled Young Migrants: Evidence from the Zhejiang Province of China

**DOI:** 10.3390/ijerph20010616

**Published:** 2022-12-29

**Authors:** Xizan Jin, Hongfei Yu, Fangxin Yi, Lili Chen, Song Wang

**Affiliations:** 1 Chinese Academy of Housing and Real Estate, Zhejiang University of Technology, Hangzhou 310023, China; 2 School of Management, Zhejiang University of Technology, Hangzhou 310023, China; 3 School of Social and Behavioral Science, Nanjing University, Nanjing 210023, China; 4 Innovation Center for Risk Governance, School of Social Development and Public Policy, Beijing Normal University, Beijing 100875, China

**Keywords:** housing unaffordability, migration, highly skilled young migrants, land provision

## Abstract

Many studies have concluded that, since housing pressure affects the mobility of highly skilled young migrants (HSYMs) in Chinese cities and regions, it is necessary to apply corresponding housing policies to adjust housing unaffordability for HYSM. This study uses data from a survey conducted in China’s Zhejiang Province, where specific policies have been implemented to attract talent. We found that housing crowds out HSYM from a city, but that the HSYM who have a master’s degree or above, or who work in government organizations or state-owned enterprises, are more tolerant of housing unaffordability. Those who are unmarried or those staying in the city for a long period are less tolerant of housing unaffordability. Meanwhile, different factors have heterogeneous impacts on the HSYMs’ tolerance for housing unaffordability across cities of different levels. Therefore, housing policies should highlight urban differences and intra-group differences, and more housing land should be provided to attract talent.

## 1. Introduction

Since the commoditization reform of housing in China in 1998, housing prices have surged from CNY 2063 per square meter in 1998 to CNY 9860 in 2020. From 2010 to 2018, housing prices in major cities including Beijing, Guangzhou, and Shenzhen had an average annual growth rate of above 20%, triggering the widespread relocation of young people who left Beijing, Guangzhou, and Shanghai to avoid higher housing costs. At the same time, the growing aging population and the decreasing birth rate pose major threats to local governments’ efforts to promote the urban economy, due to the lack of human capital. Therefore, housing unaffordability has become a significant urban issue for local governments aiming to promote the economies of first-tier cities. In order to solve the problem of youth population loss, first-tier cities in China have implemented preferential housing policies to attract highly skilled young migrants (HSYMs) (The “highly skilled young migrants” are those who have a college degree or a higher level of education, because education has a marked effect on economic and political outcomes This article defines the group of HSYMs as being under 35 years old in Zhejiang Province. They are not native residents of Zhejiang but are employed in cities in that province) Compared with other countries that pay more attention to the equity of the housing unaffordability [1,2], local governments in China prefer to use HSYM housing subsidies and hukou preferential policies, based on education and skill levels, to attract talent to their areas [3].

This article on housing unaffordability aims to answer the questions of who has been crowded out by unaffordable housing prices in destination cities, and who continues to migrate to these cities. Relevant studies have shown that housing unaffordability lessens the attractiveness of megacities for highly educated individuals [4,5,6]. Housing has become the biggest expenditure for HSYMs and thus plays a determinant role in migration decisions [7]. As a household’s primary expenditure, housing costs also play a significant role in differences in the skill composition of workers across cities. Higher housing prices are more likely to push out low-skilled workers than highly skilled workers [8,9]. According to the push–pull theory, the matter of whether laborers ultimately choose to flow into or out of a particular place is the balanced result of a pulling force and a pushing force, and housing price is an essential factor in this dynamic [10,11]. Changes in urban housing costs break the urban spatial equilibrium, and lead to a series of economic responses, such as changes in employment, wages, urban facilities costs, and land prices [12]. In China, Chen et al. [13] attempted to elucidate how housing unaffordability determines the demographics of migrants; their results show that the attractiveness of superstar cities has declined over time as housing becomes increasingly unaffordable. However, they fail to analyze the heterogeneity of HSYMs and the relationship between the individual characteristics of these groups and their housing unaffordability preferences.

This article draws the following conclusions. First, the empirical results show that housing unaffordability crowds out HSYMs from a city. Second, there are group differences in terms of housing unaffordability for HYSMs. HSYMs who have a master’s degree or above, or who work in government organizations or state-owned enterprises, are more tolerant of housing unaffordability. However, unmarried or long-term city residents are less tolerant of housing unaffordability. Finally, the authors find that certain factors have heterogeneous impacts on HSYMs’ tolerance for housing unaffordability across cities of different levels. This article makes the following contributions to the existing literature. First, it contributes to the literature on housing unaffordability and location choice by investigating the factors that influence HSYMs’ tolerance for housing unaffordability [13]. Second, we explore the heterogeneity of housing unaffordability and how these heterogeneities manifest in sub-provincial and prefecture-level cities. Third, the findings suggest that policy implications vary by city and by group. The governments of cities that are more economically developed and have higher political statuses should assign more land for building shared ownership homes (SOHs). At the same time, SOHs should be provided to unmarried HSYMs who are long-term city residents.

The remainder of this article is structured as follows: Section 2 reviews housing policies introduced in major Chinese cities, migration studies in other countries, especially in Central and Eastern Europe, and the factors that affect housing unaffordability for HYSMs; Section 3 describes how an index is built to measure housing unaffordability, and introduces the empirical model, and details the data sources, variables, and practical design. Section 4 presents the main empirical results. Section 5 concludes the study and discusses the policy implications.

## 2. Background

China’s major cities began to “compete for talent” from 2017 onwards, with many cities, such as Chengdu, Hangzhou, Chongqing, Wuhan, Xi’an, Tianjin, Nanjing, Zhengzhou, and Changsha, introducing policies to attract urban talent. “New first-tier cities” have successively introduced new talent policies to attract college students and professional and technical personnel [14]. For example, the “Excellent Talent Residency Plan” in Beijing, the “Five-year Retention of One Million College Students” in Wuhan, the “Million Talent Plan” in Hainan, the “Haihe Talent” in Tianjin, and the “Innovative Talents Express Entry Policy” in Hong Kong, have been implemented to attract HSYMs. The most commonly used policy tool in these plans is the household registration (*hukou*) system, and housing subsidies for the HSYMs (see Appendix A). Internationally, because of the diffusion of points-based systems, policies facilitating the entry of skilled and highly skilled migrants were particularly popular in the 2000s [15]. Canada and Australia introduced points-based system. Since the beginning of the 21st century, European governments have failed to attract enough skilled immigrants, so the points system has been widely adopted in Europe. For example, Denmark created its Green Card Scheme in 2007 and the Netherlands implemented a scoring system in 2008 [16]. In addition, European municipal authorities influence their dynamic and structural policies by creating a comfortable urban environment and housing, increasing transportation convenience, promoting the employment of local university graduates, and using other soft measures to attract high-quality talent [17].

Many countries from around the world have carried out extensive research on migration from the perspectives of population, socio-economy, and spatial layout. Housing price is an important factor that affects migration. Berger analyzed American census data since 1980 and concluded that migrants are more reluctant to choose cities with high housing costs [18]. Plantinga et al. reached the same conclusion when their research samples were refined to American men in full-time employment aged 35 to 65, and college-educated American men [19]. Although research assessing housing unaffordability in the context of western, developed countries’ housing policies has dominated the field, an increasing number of such studies are emerging from other countries [20,21,22]. Most of these studies follow the framework of Roback [23], which describes factors such as income, housing cost, and amenities, which determine people’s migration decisions.

High housing prices that deter immigrants and lead to the loss of human capital have always been the topic of heated discussions. In general, higher housing costs reduce the likelihood of people choosing to live in a specific location within a city [19]. Rising house prices have a crowding-out effect on migrants as they reduce the affordability of housing [24]. Early research on the impact of housing costs on migration in Western countries found that high housing prices have a negative impact on immigration [18,25,26]. Many studies argue that high housing prices directly lead to labor outflow [27]. Monk [28], and Rabe et al. [7] conclude that relatively high housing prices constrain cross-regional labor inflows, which is the main reason for labor shortages in the southeast regions of the UK. Additionally, Smith and Sage found that the sharp rise in house prices had squeezed out young people in the UK to a significant degree [29]. Brakman et al. [30] reached a similar conclusion using data from German sub-regions. However, Germany’s migration pattern has shifted from a period of reurbanization, characterized by movement up the urban hierarchy (2006–2012), to a new period of suburbanization [31,32,33,34] (since 2015). Rising housing prices are pushing people out of cities to inland and rural areas [35]. Foote [36] points out that rising housing prices have a positive wealth effect for homeowners but a negative locking effect on labor migration decisions.

In Moscow, the effect of housing prices on internal migration is very limited. Internal migration is more strongly influenced by other factors, such as distance from work, educational facilities, and the prestige of the region. Meanwhile, the significant impact of housing prices is only evident for the residence choices of highly diverse groups of international migrants. Kashnitsky found that the inflow of internal migration is generally made up of a younger population because a large number of them are students; women dominate in internal migration. International migrants are, on average, older, and men are the dominant demographic [37].

Helpman [38] introduced the housing market factor to Krugman’s [39] new economic geography and pointed out that a region’s high housing prices diminish laborers’ relative utility, which inhibits labor aggregation. After that, many scholars carried out empirical tests on Helpman’s theoretical analysis [7], consistently confirming that housing prices are the market mechanism of population migration induced by decentralized forces. However, using researchers as objects, Lin et al. [40] found that increasing housing prices are more attractive to professionals across 51 Chinese cities. Therefore, high housing prices have a strong inhibitory effect on the migration of young people, and the phenomenon of the suburbanization of immigrants can be seen in some western countries. In China, however, this phenomenon is not prominent, especially for highly educated and talented groups.

Furthermore, from a micro perspective, housing is closely related to individual utility and urban social utility. Housing accessibility and unaffordability affects the urban choices of laborers by influencing family wealth, family consumption, children’s education levels, and personal salary levels. The theory of spatial equilibrium holds that changes in productivity in the local labor market will lead to changes in employment, wages, and price levels, which will in turn affect labor welfare [12]. The rise in house prices increases the cost of living, which leads to a decrease in real income, thus inhibiting consumption [41,42]. Housing can also affect parents’ income and education levels, thus reproducing social inequality [43].

HSYMs constitute a specific labor group. The first reason for this is that this group is distinct from the general labor population because they are in the early stages of their lives. For young laborers, housing preferences and opportunities are strongly related to life events including marriage, fertility, and changes in job positions [44,45]. The timing of these events also restricts housing demand and unaffordability [44]. Second, the spatial sorting literature divides labor into the categories of high skilled and low skilled [9]. Regional differentials in human capital agglomeration, skill-based compensation, cost-of-living, amenities, and the like, lead cities of different sizes to attract laborers with different skill levels; low-skilled migrants are found to have little incentive to co-locate with highly skilled workers [9]. Studies on migration in Europe also show that highly skilled talent are more likely to settle in high-income urban areas with high urban vitality [46]. Their success in migration depends largely on their cultural capital before migration (education, work experience, language knowledge and general and specific skills) [47]. Among them, education is the key factor for the success of skilled talent [48]. A highly skilled worker can benefit marginally more from congested cities through learning and knowledge spillover, and is thus more tolerant of high housing costs [49]. The cost of living in the place of inflow affects population movement and agglomeration. The rising cost of housing significantly reduces the willingness of laborers to flow into cities [50], and its inhibitory effect is more pronounced for the influx of low-educated laborers, young laborers, and female laborers [51].

In recent years, with rising housing prices in China, housing also has an investment value, which affects class mobility, especially for HYSMs [52,53]. There is a conflict between the expected social contributions of young people and their anxieties regarding overcrowded housing or high housing prices in cities, which directly impacts their migration decisions. As a consequence, there are numerous studies on the relationship between housing and labor mobility in China. However, what distinguishes the study of China’s housing system the country’s special household registration (*hukou*) system (The hukou system was first set up in cities in 1951 and extended to rural areas in 1955. It is a household-based population management system, and it strictly controlled the population flow between regions. [54].), which distinguishes China’s urban and rural areas. China’s labor mobility research also includes rural-to-urban labor migration and interprovincial regional labor mobility research.

Rural-to-urban migrants are often characterized as laborers with low levels of education and income, while interprovincial regional migrants usually have a relatively high level of education and cultural status, are highly skilled, and have high incomes, with a strong desire to live in the city [55]. Gao investigated panel data from 35 large and medium-sized cities in China from 2000 to 2009 and found that rising housing prices in cities induce labor outflows, and this inhibition is reflected in rural labor [56]. This inhibition effect has not eliminated the continued inflow of people from other areas, and Fan et al. concluded that the new migrating population mostly comprises low-skilled laborers, mainly living in low-cost housing, and residential transactions are not closely related [57]. However, the rapid rise in prices in China’s first-tier cities not only has spillover effects on low-skilled laborers, but also makes first-tier cities less attractive to highly skilled laborers [13]. As the housing pressure on first-tier cities grows, the outflow trend becomes apparent, due to the high cost of all economic activities caused by rising housing prices [11].

However, the impact of housing stress on housing choices is moderated by other factors, such as individual utility [58,59], and social utility [60,61,62]. Further analysis shows that there are group differences in terms of the negative impact of housing unaffordability. For those who do not own their own homes, the high cost of living increases the probability of moving at some point. Wen [63] found that the effect of house prices on workers’ willingness to stay in cities with different skills, incomes, mobility, and ages is heterogeneous. In addition, for young people, housing preferences and opportunities are highly related to life events such as marriage, having children, and changes in job positions [44,45]. For decades, inland cities in Central and Eastern Europe have been characterized by physical decay, ageing in place, and selective eviction. In Polish and Czech second-order cities there are “transitory urbanites”: young families, most of whom choose to live in the city center, but with high levels of mobility [64].

The literature on labor mobility generally acknowledges the growing trend of population concentrations in large cities and metropolitan areas [65,66,67,68]. Laborers make decisions to move to big cities for higher expected incomes and a better life, better jobs, high-quality education, and a lively social environment; therefore, housing is a critical factor of individual and social utility [69]. These studies show that different cities have varied development opportunities for young people. Regional economic activities also lead to cities of different sizes attracting laborers with different skill levels [9]. Existing studies have shown that HSYMs prefer to go to first-tier cities. Therefore, HSYMs in different cities will tolerate different housing situations.

## 3. Method

### 3.1. Data

The data used in this article are from a survey that was conducted by the Zhejiang Housing Provident Fund Center and the Zhejiang University of Technology in April 2018. The object of this survey was to investigate the permanent resident population, aged 16 to 60, who have lived in urban areas for more than 6 months. They are not native residents but have been employed in cities in Zhejiang Province. The survey of this article covered the 24 counties (urban area) in 11 cities in Zhejiang Province, including Hangzhou, Ningbo, Wenzhou, Jiaxing, Huzhou, Shaoxing, Jinhua, Quzhou, Zhoushan, Taizhou, and Lishui. A detailed survey was conducted on group characteristics, housing conditions, housing needs, constraints to solving housing problems, and housing provident fund support measures, and a total of 10,337 valid survey samples were obtained. In the final analysis, this article took samples of individuals with a college degree or above and under the age of 35 as focus of this research object, and the analysis sample size was 2724.

### 3.2. Variables

This article focuses on HSYMs who lived in a city in Zhejiang Province during the survey period; it investigates the factors that influence their tolerance for housing unaffordability. Table 1 shows each variable’s statistical description. Will is the dependent variable that indicates the long-term residence intentions of HSYMs. The following question measures their willingness to stay in the city: “How long do you plan to live/work in the city in the future?”. This article considers those who choose to stay for five more years as being willing to live there long-term, and Will = 1; otherwise, Will = 0. Existing studies generally use five years as the standard for defining the long-term willingness to stay of the migrant population in China [70]. Moreover, many policy designs in China use five years as the minimum threshold. For example, in many cities, including those in Zhejiang, the condition for young talented professionals to buy new homes with price discounts is to hold and stay in the homes for at least five years before reselling. See Section 3.1.

This article defines housing unaffordability as the ability of households to purchase housing within their income levels [71]. Tolerance of housing unaffordability refers to individual tolerance for the unaffordability of housing prices and is also the individual’s willingness to live in the city in the long term. If they cannot tolerate the house prices in their city, they will move out of the city where they currently live. Although the housing price to income ratio (PIR) is commonly used to reflect the existence of a housing bubble from the perspectives of housing unaffordability and housing market sustainability [72], this article uses the PIR at the individual level to measure housing unaffordability at the individual level. The housing price income ratio PIR is calculated according to Equation (1). It is the ratio of the average housing price of the HSYMs’ city k (AP_k_) to his/her annual household income after tax in the year before the survey (Income_i_). The coefficient of PIR, as well as that of PIR×PIR, measures the HSYMs’ housing unaffordability. Housing unaffordability refers to the housing cost burden that an HSYM has to bear in the city:

(1) $${\mathrm{PIR}}_{i}=\frac{{\mathrm{AP}}_{k}}{{\mathrm{Income}}_{i}}$$

Non-housing-affordability factors are considered, which may influence the willingness of the HSYMs to reside in the city in the long term. As argued by Yang et al., the impact of housing prices on city choice by HSYMs is heterogeneous in terms of individual, family, and economic characteristics, as well as life cycles and occupations [73]. This article includes four categories of variables, which are specified and justified as follows (for the specific definitions, see Appendix B):

(1) Demographic characteristic variables. Gender, age (Age), education level (Edu), marital status (Unmarried), and family population (Member) are factors that influence migrants’ resettlement decisions [74].
(2) Employment variables. Different jobs in terms of enterprises’ ownership status (Occupation) and industry types (Industry) and different labor skills will affect the migration of the HSYMs between cities [53].
(3) Migration history variables. Migration history variables include the period of staying (POS), which is the number of years the HSYM’s family has lived in the city, and the mobility mode (MM), which reveals whether the HSYM moved from another urban area or from a rural area to the current city.
(4) Housing condition variables. This is a vector of variables, including access to a housing provident fund (HPF) and homeownership status (HOS).

This article also considers the heterogeneity of intolerance for housing unaffordability across cities of different economic scales and positions on the political hierarchy. The authors group the 11 cities in Zhejiang Province into two categories, sub-provincial and prefecture-level cities. Hangzhou and Ningbo are sub-provincial cities, and the other cities are prefecture-level cities.

### 3.3. Empirical Design

Given that willingness to be a long-term resident is the dependent variable and a dummy variable, the authors built a binary logistic model, as shown in Equation (2):

(2) $$\begin{array}{cc} \mathrm{Logit}({\mathrm{Will}}_{i})= & {\mathrm{Con}+\alpha }_{1}{\mathrm{PIR}}_{i}{+\alpha }_{2}{\mathrm{PIR}}_{i}{\times \;\mathrm{PIR}}_{i}{+\beta }_{1}{\mathrm{Gender}}_{i}{+\beta }_{2}{\mathrm{Gender}}_{i}{\times \;\mathrm{PIR}}_{i}\\ & \;{+\gamma }_{1}{\mathrm{Edu}}_{i}{+\gamma }_{2}{\mathrm{Edu}}_{i}{*\;\mathrm{PIR}}_{i}{+\delta }_{1}{\mathrm{Unmarried}}_{i}\\ & \;+{\;\delta }_{2}{\mathrm{Unmarried}}_{i}{\times \;\mathrm{PIR}}_{i}+\mu_{1}{\mathrm{Occupation}}_{i}\\ & \;+{\;\mu }_{2}{\mathrm{Occupation}}_{i}{\times \;\mathrm{PIR}}_{i}+\vartheta_{1}{\mathrm{Industry}}_{i}+\vartheta_{2}{\mathrm{Industry}}_{i}{\times \;\mathrm{PIR}}_{i}\\ & \;{+\rho }_{1}{\mathrm{MM}}_{i}{+\rho }_{2}{\mathrm{MM}}_{i}{\times \;\mathrm{PIR}}_{i}{+\rho }_{1}{\mathrm{POS}}_{i}{+\rho }_{2}{\mathrm{POS}}_{i}{\times \;\mathrm{PIR}}_{i}\\ & \;{+\tau }_{1}{\mathrm{HPF}}_{i}{+\tau }_{2}{\mathrm{HPF}}_{i}{\times \mathrm{PIR}}_{i}{+\varphi }_{1}{\mathrm{Age}}_{i}{+\omega }_{1}{\mathrm{Member}}_{i}+\mathrm{City}+\in_{i} \end{array}$$

$\alpha_{1}$ and $\alpha_{2}$ are coefficients of ${\mathrm{PIR}}_{i}$ and ${\mathrm{PIR}}_{i}\times {\mathrm{PIR}}_{i}$. The two coefficients measure the tolerance for housing unaffordability by HSYMs. This article includes the interaction terms between ${\mathrm{PIR}}_{i}$ and the eight categories of variables (${\mathrm{Gender}}_{i}\times {\mathrm{PIR}}_{i}$,${\;\mathrm{Edu}}_{i}\times {\mathrm{PIR}}_{i}$,${\;\mathrm{Unmarried}}_{i}\times {\mathrm{PIR}}_{i}$,${\;\mathrm{Occupation}}_{i}\times {\mathrm{PIR}}_{i}$,${\;\mathrm{Industry}}_{i}\times {\mathrm{PIR}}_{i}$,${\;\mathrm{MM}}_{i}\times {\mathrm{PIR}}_{i}$,${\;\mathrm{POS}}_{i}\times {\mathrm{PIR}}_{i}$, ${\mathrm{HPF}}_{i}\times {\mathrm{PIR}}_{i}$), and their coefficients ($\beta_{2}$, $\gamma_{2}$, $\delta_{2}$, $\mu_{2}$,$\;\vartheta_{2}$, $\rho_{2}$, $\sigma_{2}$ and $\tau_{2}$). It measures the adjustment effects of the eight categories of variables on the HSYMs’ tolerance for housing unaffordability.${\;\mathrm{Age}}_{i}{\;\mathrm{and}\;\mathrm{Member}}_{i}$ are two continuous variables. City indicates the city fixed effect, Con represents the constant term, and $\epsilon_{i}$ is the error.

## 4. Results

This section details three features of the empirical results. In Section 4.1., this article demonstrates that housing unaffordability, education level, industry type, marital status, the mobility mode, period of stay, and home ownership all significantly impact HSYMs’ willingness to reside in a city long term. In Section 4.2., this article discusses the factors that affect the HSYMs’ tolerance for housing unaffordability. In Section 4.3., we demonstrate the heterogeneous effects across cities regarding HSYMs’ tolerance for housing unaffordability.

### 4.1. Factors That Affect Long-Term Residence Willingness of the HSYMs

In Table 2, the dependent variable is long-term residence willingness (Will). Column (1) shows that the coefficient of the housing unaffordability (PIR) is −0.628 and the significance is at the 1% level, which means the higher the PIR is, the lower the willingness of an HSYM to settle down in the city for an extended period. The coefficient of PIR×PIR is 0.0412 and significant at the 5% level, which indicates that the reduction in long-term residence willingness is associated with an increase in PIR, but that higher PIR will lead to an increase in long-term residence willingness.

In Column (2), the authors further control demographic characteristics and their interaction terms with PIR. From Column (3) to Column (5), we add the categories of variables one by one, including employment factors, migration history, and housing conditions, as well as their interactions with PIR. The results show that the coefficients of most variables are consistent across the columns and the Pseudo R2 in Column (5) reaches 0.1661. Thus, the results in Column (5) are robust. As shown in Column (5), the coefficient of housing unaffordability (PIR) is −0.806 and is significant at the 1% level, and the coefficient of PIR×PIR is not significant.

The coefficient of education level (Edu) is 0.446 and is significant at the 10% level, indicating that the long-term residence willingness of the HSYMs with a master’s degree or above is 56.21% (e^0.446^ − 1) higher than that of the HSYMs with a college or bachelor’s degree. This indicates that the sample cities are more attractive to highly skilled young migrants. The coefficient of marital status (Unmarried) is −0.457 and is significant at the 1% level. This indicates that unmarried HSYMs’ long-term residence willingness is 36.7% (1 − e^−0.457^) lower than that of married or divorced HSYMs, given the other variables controlled. Without family burdens, unmarried people tend to be more mobile and able to pursue better opportunities across cities.

In terms of employment factors, the long-term residence willingness of the HSYMs working in labor-intensive industries (Industry) is 29.56% (e^0.259^ − 1), which is higher than those working in non-labor-intensive industries. This can probably be explained by the fact that labor-intensive industries are quite similar across cities. Migrating to another city does not improve the HSYMs’ welfare.

The coefficient of mobility mode (MM) is −0.302 and is significant at the 10% level, which means that the long-term residence willingness of the HSYMs who have moved from another urban area to the current city is 26.07% (1 − e^−0.302^), lower than the HSYMs who are from rural areas. This indicates that the urban-born HSYMs are more mobile than their rural-born counterparts.

The period of staying (POS) is 0.302 and is significant at the 1% level, which means that the longer the HSYMs have been staying in a city, the more likely they are to be willing to live in that city in the long term. Homeownership (HOS) also significantly influences long-term residence willingness. Its coefficient is 1.008 and is significant at the 1% level, which means owning a home in the city is associated with long-term residence willingness that is higher by 174.01% (e^1.008^ − 1), with other variables controlled. This can be explained by the fact that, if people own a home in a city or if they have been there for longer, they may have a deeper attachment to the city.

### 4.2. Factors That Have Adjustment Effects on Tolerance for Housing Unaffordability

#### 4.2.1. Demographic Characteristics

As shown in Column (5) of Table 2, the coefficient of the interaction term between PIR and gender is not significant at 1%, which means tolerance for housing unaffordability is not affected by gender differences. The coefficient of the interaction term between PIR and Edu is 0.524 and is significant at the 1% level. This means the HSYMs who have a master’s degree or above have a higher tolerance for housing unaffordability than those with bachelor or other college degrees. This is mainly because HSYMs with higher education levels have more sophisticated knowledge and better learning skills, meaning that they benefit more from knowledge spillover through a higher level of agglomeration in a city with higher housing prices. This results in their higher tolerance for the current housing unaffordability. The coefficient of the interaction term Unmarried*PIR is −0.340 and is significant at 1%, which indicates that the unmarried have a lower tolerance for housing unaffordability. The unmarried are more mobile than the married, and they are more able to be flexible and migrate to a city that can give them better welfare (lower housing burdens, higher income, better amenities).

#### 4.2.2. Employment

The coefficient of the interaction term Occupation × PIR is 0.545 and is significant at 1%. This indicates that HSYMs working in government organizations and state-owned enterprises have a higher tolerance for housing unaffordability than the HSYMs working in non-government organizations and non-state-owned enterprises. This can be explained by the difference in occupation stability between the two types of enterprises. In China, jobs in government organizations and state-owned enterprises are tenured, and HSYMs working in these organizations are less likely worried about unemployment. They are regularly paid and can often receive generous bonuses. In this way, they have less pressure and face lower income risks than those in private organizations and have a significant tolerance for housing unaffordability. In contrast, the interaction term between the tolerance for housing unaffordability (PIR) and industry type (Industry) is not significant. This means that the industry status (Industry) of HSYMs did not have a significant effect on their tolerance for housing unaffordability.

#### 4.2.3. Migration History and Housing Conditions

As shown in Column (5) of Table 2, the interaction term between the mobility mode and tolerance for housing unaffordability (MM × PIR) is not significant. However, the interaction term between the period of staying and the tolerance for housing unaffordability (POS × PIR) is −0.274 and is significant at the 5% level. This indicates that a longer period of residence reduces tolerance for housing unaffordability, which can be explained by the fact that, if HSYMs’ income cannot keep up with the city’s economic development and rising housing prices, they cannot afford to buy a home in the city while they reside there. They lose their patience and the hope of owning a home in the city, and thus have a lower tolerance for housing unaffordability. The coefficient of HPF × PIR and HOS × PIR for HSYMs is not significant. This means that owning a home (HOS) and having housing providence do not affect HSYMs’ tolerance for housing unaffordability. This may be because the housing loan policy in the province as a whole causes similar housing pressures for both homeowners and renters, and the housing policy subsidies are insufficient.

In summary, this article finds the HSYMs who have a master’s degree or above, or who work in government organizations or state-owned enterprises, are more tolerant of housing unaffordability. However, the unmarried or those who have resided in the city for a long period are less tolerant of housing unaffordability.

### 4.3. Heterogeneity Effects between Sub-Provincial Cities and Prefecture-Level Cities

The authors expect these factors to have heterogeneous adjustment effects on tolerance for housing unaffordability among HSYMs across cities with different economic statuses and positions on the political hierarchy. The 11 sample cities in Zhejiang Province are grouped into two categories. Sub-provincial cities have higher levels of economic development, higher agglomeration levels, and higher housing prices than the other category: prefecture-level cities. The results are provided in Table 3.

#### 4.3.1. Heterogeneity in Adjustment Effects of Demographic Characteristics on Tolerance for Housing Unaffordability

Comparing the results in Column (1) to those in Column (2) in Table 3 shows the heterogeneous adjustment effects of Gender, Edu, and Unmarried on HSYMs’ tolerance for housing unaffordability. The interaction term Gender × PIR is not significant in Column (1) but is negative with 1% significance in Column (2). This means that, in sub-provincial cities, there is no difference in the tolerance for housing unaffordability between male and female HSYMs; however, in prefecture-level cities, compared to female HSYMs, male HSYMs have less tolerance. This could be explained by the fact that male HSYMs are more mobile. They leave for more developed sub-provincial cities if they are unsatisfied with their lives in a prefecture-level city with worse housing unaffordability.

The coefficients of the interaction term Edu × PIR in the two columns show that the more highly educated and higher-skilled HSYMs in sub-provincial cities have a higher tolerance for housing unaffordability, compared to their relatively lower educated and lower-skilled counterparts. However, there is no difference between the two groups of HSYMs in prefecture-level cities. This can be explained by the fact that sub-provincial cities are highly concentrated, with more highly educated HSYMs benefiting more from the knowledge spillover of agglomeration, which offsets more of the adverse effects of housing unaffordability. However, in the prefecture-level cities where there is a relatively low level of agglomeration, the more highly educated experience less knowledge spillover due to the lower levels of agglomeration.

The coefficients of the Unmarried × PIR in the two columns show that unmarried HSYMs in the sub-provincial cities have a lower tolerance for housing unaffordability compared to their married counterparts. However, there is no difference between the two groups of HSYMs in prefecture-level cities. The difference between the two groups of cities can be explained by the fact that the unmarried are more mobile and can easily choose to move from sub-provincial cities to prefecture-level cities to reduce their housing burden, because the housing prices in the sub-provincial cities are much higher than in prefecture-level cities. However, for the HSYMs in prefecture-level cities, moving across to other prefecture-level cities does not significantly help in reducing their housing burdens.

#### 4.3.2. Heterogeneity in Adjustment Effects of Employment on Tolerance for Housing Unaffordability

The coefficients of the interaction term Occupation × PIR in the two columns show that, in sub-provincial cities (Column 1 in Table 3), the HSYMs working in government organizations and state-owned enterprises have a slightly higher tolerance for housing unaffordability compared to that of HSYMs in non-government organizations and non-state-owned enterprises, which indicates that Occupation has low adjustment effects. Similarly, in prefecture-level cities (Column 2 in Table 3), Occupation also has a significant moderating effect on housing unaffordability, and it is even more significant in sub-provincial cities. The differences between the two groups of cities can be explained by the fact that government organizations and state-owned enterprises in prefecture-level cities, where resources are limited, provide far better benefits and more stability to HSYMs compared to other (privately owned) enterprises. However, these advantages of government organizations and state-owned enterprises is minimized in sub-provincial cities where economic development is higher and social-economic resources are more readily available for privately-owned enterprises. As a result, enterprises of both ownership statuses can provide similar levels of income and stability to HSYMs in sub-provincial cities, and the enterprise owner does not have high adjustment effects on the HSYMs’ tolerance for housing unaffordability.

#### 4.3.3. Heterogeneity in Adjustment Effects of Migration History on Tolerance for Housing Unaffordability

In Columns (1) and (2) in Table 3, the coefficients of POS × PIR show that, in the sub-provincial cities (Column 1 in Table 3), the HSYMs who have resided in the city for a longer period have a lower tolerance for housing unaffordability. However, the adjustment effect of POS is not significant in the prefecture-level cities. The different adjustment effects of POS between the two groups of cities can be explained by the fact that it is more challenging to climb up the housing ladder in sub-provincial cities, where housing prices are much higher, than in prefecture-level cities. It is difficult for HSYMs in sub-provincial cities to match their incomes with housing price growth. They are therefore more likely to leave the area due to the increasing housing pressure. However, in prefecture-level cities, even if the housing pressure increases, the income level of the HSYMs can keep up with these rises, and the overall consumer price level remains much lower than that in sub-provincial cities.

#### 4.3.4. Heterogeneity in the Adjustment Effects of Housing Condition on Tolerance for Housing Unaffordability

The results show that homeownership (HOS) has heterogeneous adjustment effects in cities of different economic statuses and at different positions on the political hierarchy. In the sample of sub-provincial cities (Column 1 in Table 3), the coefficient of the interaction term HOS × PIR is 0.728 and is significant at the 1% level, which indicates that an HSYM who owns a home in a sub-provincial city has a higher tolerance for housing unaffordability. In contrast, the coefficient of HOS × PIR for the HSYMs in prefecture-level cities (Column 2 in Table 3) is not significant, which means that owning a home for an HSYM in a prefecture-level city does not generate higher tolerance for housing unaffordability. This heterogeneity effect can be explained by the fact that the cost of buying and renting a house is relatively low, so it is easier to own a home in prefecture-level cities compared to sub-provincial cities, where housing prices are much higher, and owning a home creates a greater attachment for an HSYM to the local city. Thus, owning a home in sub-provincial cities raises the HSYMs’ willingness to stay in the city compared to HSYMs who do not own a home. Although these homeowners still face high housing costs, they want to climb the housing ladder.

## 5. Conclusions

This article investigates the adjustment effects of factors on the impacts of housing unaffordability (PIR) on HSYMs’ long-term residence willingness, showing how these factors influence HSYMs’ tolerance for housing unaffordability in a city. The authors offer the following three findings. First, as is consistent with most existing studies [41,42,43,44], this article finds that housing unaffordability (PIR), demographic characteristics, employment factors, migration history, and housing condition factors influence the long-term residence willingness of HSYMs in a city.

Second, this article enriches existing housing-unaffordability-related literature, and it provides several new perspectives on the adjustment effects of some non-housing-affordability factors on HSYMs’ tolerance for housing unaffordability. Specifically, HSYMs who have a master’s degree or above, or who work in government organizations or state-owned enterprises, are more tolerant of housing unaffordability. However, the unmarried or those who have resided in the city for a long period are less tolerant of housing unaffordability.

Third, this article demonstrates the heterogeneity effects across cities of different economic and political statuses regarding the adjustment effects of non-housing-affordability factors on HSYMs’ tolerance for housing unaffordability. The sub-provincial cities are more economically developed, have higher statuses on the political hierarchy, and have more abundant social and economic resources, but also have higher housing prices than prefecture-level cities, which leads to the following heterogeneous adjustment effects. In sub-provincial cities, higher education and more stable jobs still have a positive effect on housing unaffordability, while being single and living in a city for a long time have a negative effect; these findings are consistent with the overall conclusion. However, in prefecture-level cities, only employment had a significant effect on housing unaffordability. These results suggest that housing unaffordability for HYSMs working in first-tier cities is more susceptible to other factors, while in prefecture-level cities, jobs are the single most important factor.

## 6. Policy Implications

Housing policy has a huge impact on the motivations for migration. In countries such as Europe and the US, some young people prefer renting or relocating to small and medium-sized cities rather than purchasing houses, due to the high prices. The research finds that highly skilled young migrants also encounter housing unaffordability issues, which negatively influences their willingness to purchase houses. Therefore, it is important to make efforts to readjust Chinese housing policy for the younger generations. China has a high homeownership rate (73.85%, according to the seventh census in 2020) and home equity share, compared to other countries such as those in Europe. The 2019 Survey on Household Assets and Liabilities of Urban Residents in China, released by the People’s Bank of China, shows that, in a survey of 30,000 households nationwide, the average total household asset is 3.179 million yuan. Yet, 70% of these assets are houses, with a much higher property share than the 34.6% in the US. Since the development of China’s commodity market in 1998, housing prices have been rising. The growth of housing prices sometimes far exceeds the growth of residents’ incomes, resulting in increasing pressure on residents’ housing consumption.

Thus, controlling the overheated real estate market can reduce the crowding-out effect of excessive housing prices on highly skilled young migrants. Relatedly, controlling housing prices also helps to improve housing affordability. This can effectively ensure the retention of HSYMs in cities and avoid the loss of a large number of highly qualified people. Therefore, we put forward some policy suggestions: continue to use real estate policy to stabilize housing prices and reduce the living pressures caused by high housing prices; sustain the reasonable regulation of regional land supply to increase the supply of affordable housing; increase the supply of talent housing for young professionals. For example, the governments of cities that are more economically developed and have higher statuses within the political hierarchy should assign more land for the construction of shared ownership housing (SOH). The SOH scheme in China now is a widely acceptable housing ownership method in many cities. SOH provides homeownership (or at least the right to use it) at a lower cost to home buyers. This paper finds that homeownership has a positive moderating effect on housing unaffordability tolerance in sub-provincial cities, but not in prefecture-level cities. Because of the higher housing prices in sub-provincial cities with higher levels of economic development and higher political status, it is challenging for HSYMs to own a home in the first instance, and to plan for long-term residency. Thus, SOH is well suited for addressing this issue.

Second, this research focused on highly skilled young migrants, finding not only that housing unaffordability has an impact on willingness to own housing, but also that different HSYM groups have different levels of housing unaffordability. More importantly, different factors have different effects on housing unaffordability in different grades of cities. Therefore, this article proposes that housing policies should highlight urban differences and intra-group differences. Providing additional training and further education can alleviate the adverse effects of high housing prices. The authors find that those who are better educated (with a master’s degree or above) have a higher tolerance for housing unaffordability than those who are less educated. This is because high housing prices and more housing unaffordability are associated with a higher level of agglomeration with higher population density. The better educated can benefit more from knowledge spillover from the higher level of agglomeration in cities, and thus raise their income. Furthermore, sub-provincial cities and prefecture-level cities should provide different housing assistance schemes according to the types of HSYM. Sub-provincial cities should focus on HSYMs who have lower levels of education and inflow from rural areas. They either cannot earn higher incomes or lack monetary assistance from their families, so are more sensitive to housing price growth. As such, shared ownership homes that provide home ownership at lower prices are more likely to favor these groups.

Moreover, this article finds that HSYMs who have resided in sub-provincial-level cities for longer periods have a lower tolerance for housing unaffordability. This shows that housing security should be provided to HSYMs who have lived in the city for a long time. In fact, HSYMs who have worked for a certain number of years deserve a certain degree of protection. This would make a great contribution to the sustainable development of the city. Additionally, it is an important basis for the expansion of the middle class.

In summary, this analysis of the heterogeneity of tolerance, which arises due to factors such as occupation, suggests that HSYMs tolerance to housing affordability does not predominantly stem from their current income and housing level. Rather, it arises from their expectations for the future, depending on their lifetime income. Therefore, cities need to attract and retain HSYMs. Instead of focusing on existing housing policies, they should focus on improving the sustainable advancement of young talents, by means of stable employment, security, and the sustainable development of the industry.

## Figures and Tables

**Table 1:** Statistical description of variables.

Variable	N	Mean	S.D.
Will (intend to stay = 1)	2724	0.560	0.497
PIR	2724	10,180	12,113
PIR × PIR	2724	250,360	1.285
AP	2724	10,081	3.632
Demographic characteristic variables			
Age	2724	29,050	3.478
Gender (male = 1)	2724	0.490	0.500
Edu (college and bachelor’s degree = 1)	2724	0.930	0.255
Unmarried	2724	0.440	0.497
Member	2724	2.410	1.766
Employment variables			
Occupation (government organizations and state-owned enterprises = 1)	2724	0.250	0.433
Industry (labor-intensive industries = 1)	2724	0.700	0.460
Migration history variables			
MM (migrates across urban areas = 1)	2724	0.600	0.490
POS	2724	5.450	3.232
Housing condition variables			
HPF (having a housing provident fund = 1)	2724	0.800	0.398
HOS (having housing ownership = 1)	2724	0.440	0.497

**Table 2:** HSYMs’ willingness to reside in a city long term, and factors influencing their tolerance for housing unaffordability.

	Will				
	(1)	(2)	(3)	(4)	(5)
PIR	−0.628 ***	−0.473	−0.726 **	−0.927 ***	−0.806 ***
	(0.169)	(0.326)	(0.329)	(0.304)	(0.304)
PIR × PIR	0.0412 **	0.0148	0.014	0.006	−0.021
	(0.020)	(0.022)	(0.030)	(0.035)	(0.037)
Age		0.207 ***	0.209 ***	0.035	0.002
		(0.058)	(0.060)	(0.053)	(0.039)
Gender		−0.043	−0.057 *	0.0017	0.056
		(0.033)	(0.031)	(0.035)	(0.053)
Gender × PIR		0.055	−0.058	−0.058	−0.132
		(0.149)	(0.135)	(0.153)	(0.136)
Edu		0.362 *	0.603 ***	0.410 **	0.446 *
		(0.196)	(0.219)	(0.201)	(0.250)
Edu × PIR		0.195	0.473 **	0.544 ***	0.524 ***
		(0.211)	(0.186)	(0.178)	(0.182)
Unmarried		−0.557 ***	−0.578 ***	−0.559 ***	−0.457 ***
		(0.112)	(0.109)	(0.128)	(0.116)
Unmarried × PIR		−0.126	−0.179 **	−0.302 ***	−0.340 ***
		(0.097)	(0.086)	(0.098)	(0.115)
Member		0.239 ***	0.246 ***	0.181 ***	0.087
		(0.054)	(0.058)	(0.053)	(0.055)
Occupation			0.348 ***	0.367 **	0.215
			(0.133)	(0.145)	(0.155)
Occupation × PIR			0.579 ***	0.600 ***	0.545 ***
			(0.142)	(0.139)	(0.120)
Industry			0.294 *	0.303 **	0.259 *
			(0.157)	(0.129)	(0.138)
Industry × PIR			−0.142	−0.067	0.0152
			(0.154)	(0.137)	(0.104)
MM				−0.429 **	−0.302 *
				(0.188)	(0.161)
MM × PIR				0.166	0.121
				(0.113)	(0.116)
POS				0.354 ***	0.302 ***
				(0.116)	(0.110)
POS × PIR				−0.234 **	−0.274 **
				(0.093)	(0.109)
HPF					0.485
					(0.320)
HPF × PIR					0.132
					(0.304)
HOS					1.008 ***
					(0.149)
HOS × PIR					0.180
					(0.230)
City Fixed Effect	Yes	Yes	Yes	Yes	Yes
Con	0.516 ***	0.575 ***	0.181	0.613 *	−0.457
	(0.006)	(0.192)	(0.270)	(0.317)	(0.525)
Observations	2724	2724	2724	2724	2723
Pseudo R2	0.058	0.102	0.113	0.135	0.166

Notes: standard errors are clustered at the city level, and they are presented in parentheses under the coefficients; *** *p* < 0.01, ** *p* < 0.05, * *p* < 0.1. The results in this table are based on responses from all sample cities.

**Table 3:** Heterogeneity effects across cities in terms of HSYMs’ tolerance for housing unaffordability.

	Will	
	Sub-Provincial Cities	Prefecture-Level Cities
	(1)	(2)
PIR	−1.199	−0.377
	(0.957)	(0.342)
PIR × PIR	−0.043	−0.028
	(0.042)	(0.086)
Age	−0.079	0.062
	(0.051)	(0.069)
Gender	−0.008	0.064
	(0.028)	(0.064)
Gender × PIR	0.194	−0.443 ***
	(0.261)	(0.160)
Edu	1.061 ***	−0.053
	(0.003)	(0.103)
Edu × PIR	0.502 *	0.370
	(0.277)	(0.388)
Unmarried	−0.325 ***	−0.597 ***
	(0.110)	(0.146)
Unmarried × PIR	−0.535 ***	−0.315
	(0.075)	(0.227)
Member	0.013	0.115
	(0.121)	(0.087)
Occupation	0.451 ***	0.062
	(0.100)	(0.243)
Occupation × PIR	0.399 *	0.588 ***
	(0.221)	(0.180)
Industry	0.138	0.279 *
	(0.316)	(0.168)
Industry × PIR	−0.011	0.039
	(0.125)	(0.163)
MM	0.069 ***	−0.566 ***
	(0.027)	(0.107)
MM × PIR	−0.029	0.199
	(0.158)	(0.261)
POS	0.706 ***	0.145 ***
	(0.065)	(0.029)
POS × PIR	−0.602 ***	−0.100
	(0.032)	(0.098)
HPF	1.374 ***	0.268
	(0.077)	(0.390)
HPF × PIR	0.512	−0.054
	(0.494)	(0.435)
HOS	1.037 ***	0.908 ***
	(0.048)	(0.280)
HOS × PIR	0.728 ***	−0.290
	(0.209)	(0.490)
City Fixed Effect	Yes	Yes
Con	−1.971 ***	0.261
	(0.105)	(0.160)
Observations	1040	1683
Pseudo R2	0.205	0.175

Notes: Standard errors are clustered at the city level, and they are presented in parentheses under the coefficients; *** *p* < 0.01, ** *p* < 0.05, * *p* < 0.1. The two sub-provincial cities are Hangzhou and Ningbo. The prefecture-level cities are Wenzhou, Shaoxing, Huzhou, Jiaxing, Jinhua, Quzhou, Taizhou, Lishui, and Zhoushan.

## Data Availability

Some or all of the data and models that support the findings of this study are available from the corresponding author upon reasonable request.

## Appendix A

**Table A1:** Ten cities’ talent subsidy policies in China.

Cities	Conditions	Talent Subsidy Policies
Hangzhou	New full-time university graduates with a bachelor’s degree or above who have no house and do not benefit from other housing policies in Hangzhou, and who have paid social security continuously for six months.	Each household will be paid CNY 10,000 a year for three years.
Wuhan	Students from universities within three years of graduation who have Wuhan *Hukou* and no self-owned housing in Wuhan.	Apply for a three-year college graduate rental housing.
Xi’an	Graduated students who are identified as E talents, have no house, and do not benefit from housing policies in Xi’an.	CNY 300 per month for a maximum of three years.
Nanjing	Full-time college graduates with a bachelor’s degree or above who have no house in Nanjing.	Rent subsidies: Doctorate, CNY 2000 per month; Masters, CNY 800 per month; Undergraduate (including senior engineering and above), CNY 600 per month.
Changsha	Under the age of 35, two years after graduation with a bachelor’s degree or above; have Changsha *Hukou.*	Rent and living allowance: Doctorate, CNY 15,000 per year; Masters, CNY 10,000 per year; Undergraduate, CNY per year.
Zhengzhou	Undergraduate who graduates from “Shuangyiliu” universities and master’s graduates under 35 years old; doctors of any age; no house in Zhengzhou; benefit from Zhengzhou *Hukou*.	Housing subsidies (do not have rent subsidies): Doctorate: CNY 100,000; Masters: CNY 50,000; Undergraduate: CNY 20,000.
Qingdao	Bachelor’s degree or above and have Qingdao *Hukou*.	Rent subsidies: Doctorate, CNY 1200 per month; Master’s students, CNY 800 per month; Undergraduates, CNY 500 per month.
Hefei	Undergraduate who graduated within the last three years and master’s graduates under 35 years old; doctors of any age; have Hefei *Hukou.*	Rent subsidies: Doctorate, CNY 20,000 per year; Master’s, CNY 15,000 per year; Undergraduate, CNY 10,000 per year; Graduates of higher vocational colleges, CNY 6000 per year.
Shenyang	Have Shenyang *Hukou* but originally not from Shenyang; under 35 years old; have an undergraduate, master’s, or doctorate degree.	Rent subsidies: Doctorate, CNY 1250 per month; Masters, CNY 850 per month; Bachelor, technicians, and highly skilled talents, CNY 500 per month.
Guangzhou	Local college graduates cannot apply.	Rent subsidies: Intermediate title of professional personnel, senior technicians and has a master’s degree, CNY 750 per month; Bachelor’s degree, CNY 500 per month.

## Appendix B

**Table A2:** Variable definitions.

Variable	Definition
Will	Long-term residence willingness. = 1 if respondent intends to stay in the city for a long time and has no intention of relocating; = 0 if respondent intends to stay in the city for a short time, that is, there is some willingness to relocate.
PIR	The ratio of the average housing price to the respondent’s annual household income in 2016.
PIR × PIR	The square of PIR.
AP	Average housing price in a city where HSYMs live.
Age	Age of the respondent, in years.
Gender	1 = Male; 0 = Female.
Edu	Education. 1 = college and bachelor’s degrees; 0 = master’s degree or above.
Unmarried	Marital status. 1 = unmarried; 0 = married or divorced.
Member	Number of other family members besides the head of the household.
Occupation	1 = government organizations and state-owned enterprises; 0 = non-government organizations and non-state-owned enterprises.
Industry	1 = labor-intensive industries; 0 = non-labor-intensive industries.
MM	Mobility mode. = 1 if the respondent migrates across urban areas; = 0 if the respondent migrates from rural to urban areas.
POS	Period of staying: the number of years the respondent’s family has been living in the city.
HPF	Housing provident fund. 1 = Having a housing provident fund; 0 = not having a housing provident fund.
HOS	Housing ownership. = 1 if the respondent is staying in a house owned by the respondent’s family; = 0 if the respondent is staying in a rented house.
